# Supplementary material for: Are carbon emissions trading and green financial instruments synergistic? -Comprehensive quantitative research based on content analysis
Source: PLoS One. 2024 Mar 7;19(3):e0298601. doi: 10.1371/journal.pone.0298601 (PMC10919601; doi:10.1371/journal.pone.0298601)
Supplement: S1 Data — (ZIP) [file pone.0298601.s001.zip › Data/地级市Z_F工作报告.zip!/─■╧─/╣╠╘¡╩╨/└┤╫╘╣1⁄2╓┌║┼í╛┬φ┐╦╩2╛▌═°í┐_2015.docx]

各位代表：

　　我代表市人民政府向大会报告工作，请予审议，并请政协委员和列席人员提出意见。

　　2014年工作回顾

　　一年来，市人民政府认真贯彻落实自治区党委、政府和市委的决策部署，全力稳增长、促改革、调结构、惠民生、保稳定，较好地完成了市三届人大二次会议确定的各项目标任务。

　　全市完成地区生产总值200.12亿元，增长9.3%，其中第一产业46.45亿元，增长5.6%;第二产业55.65亿元，增长16.8%(工业29.87亿元，增长14.2%);第三产业98.02亿元，增长6.8%。全社会固定资产投资300.8亿元，增长30.1%。地方公共财政预算收入15.34亿元，增长17.1%;地方公共财政支出165.1亿元，增长10.5%。招商引资到位资金134.69亿元，增长30.8%。社会消费品零售总额55.73亿元，增长12.5%。城镇居民人均可支配收入19676.5元，增长8.8%;农村居民人均可支配收入6395.3元，增长12.3%。城镇登记失业率3.87%。居民消费价格指数涨幅2.1%。人口自然增长率9.6‰。全面完成自治区下达的节能减排任务，城市空气质量优良天数达335天。在自治区效能目标管理考核的十一项经济和收入指标中，我市第二产业、工业增加值、全社会固定资产投资、地方公共财政预算收入、招商引资到位资金、社会消费品零售总额和农村居民人均可支配收入七项指标增幅居全区第一。隆德、泾源县地方公共财政预算收入突破亿元。经济社会发展呈现三大亮点：

　　——产业发展彰显特色。农业又获丰收。粮食总产84.76万吨，创历史新高。西吉县被农业部评为全国粮食生产先进单位。新增马铃薯种薯2万亩，达到28.5万亩，原原种繁育能力达到1.5亿粒，建成西北最大的马铃薯脱毒种薯繁育基地。开展基础母牛信息化管理工作，新增基础母牛5.2万头，其中引进安格斯良种母牛2900头;新增肉牛10.2万头，饲养量达到100.2万头。新增冷凉蔬菜6万亩，达到58.7万亩，全部通过无公害农产品产地认定，70%销往国内大中城市和港澳地区。新增中药材3万亩，达到13万亩。新增苗木5.2万亩，达到40万亩，建成西北重要的苗木种植基地。原州区获全区农业特色优势产业发展先进县一等奖、西吉县获二等奖，原州区获全区农业农村工作综合考评山区组第二名。启动国家农业科技园区建设。新增自治区级农业产业化龙头企业28家，各类农民合作经济组织达到1166家。农产品加工转化率达到46.3%。我市被授予中国“冷凉蔬菜之乡”和“马铃薯种薯之乡”称号，“六盘山”农产品品牌知名度进一步提升。工业再上台阶。轻工产业园一期基础设施和入园项目同步开工，盐化工示范区和各县(区)产业园区功能不断完善，新入园企业36家。全市新增规模以上企业12家，累计达到48家。金昱元盐化工一期100万吨真空制盐项目建成调试，呈祥风力塔筒制造等项目建成投产;隆德人造花工艺公司创汇1042万美元，带动就业1000多人;王洼煤矿600万吨改扩建项目进展顺利，银洞沟煤矿300万吨改扩建项目二期试生产。重点工业产品产量稳步增长，生产原煤508万吨、马铃薯淀粉14.7万吨，发电量46.2亿度。服务业健康发展。与阿联酋皇家投资局正式签订六盘山国际旅游休闲度假区项目建设合同，前期工作有序推进。完成六盘山红军长征景区改造提升工程。火石寨景区引进宁夏水洞沟旅游公司承包经营，创建为国家4A级景区。泾河源旅游小镇建设标准高，成为全区亮点。我市荣登“2014旅游业最美中国榜”，并获评“最美中国·文化旅游民俗(民族)风情目的地城市” 。固原至杭州夏季航线顺利开通。全市接待游客量和旅游社会收入分别增长15.1%和21.5%。新华百货、国芳百盛等大型商场入驻，固原宾馆、西港航空酒店等星级酒店建成运营。西兰银综合物流园公路物流港一期项目投入运营。

　　——项目建设破解瓶颈。集中开展重点项目大会战，520个投资500万元以上项目开工517个，其中100个重点项目开工97个。城乡面貌日新月异。按照“1411”城镇体系，推进区域中心城市和大县城建设。人民公园、古雁公园二期、城市规划展示馆建成开放，成为市区标志性景观。西南新区市医院迁建、九龙佳苑、安置房项目主体完成。博物馆应急避难广场、东岳山森林公园等项目开工建设。新建城市道路20条28.3公里、改造街巷79条13公里，建成公厕50座。启动“五城联创”工作，城乡环境综合整治深入推进。大县城和重点小城镇扩容提质，西吉物流园、隆德红崖老巷子二期、泾源水系景观工程、彭阳悦栖商贸中心等建成使用，四县市民休闲森林公园加快建设。推进头营、将台、平峰、联财、泾河源、王洼等6个中心镇建设，建成驼巷、单南、红崖、兴盛、韩堡等22个美丽村庄。原州区、彭阳县获全区新农村建设二等奖。西吉龙王坝村、隆德红崖村获评“中国最美休闲乡村”。城镇化率达到40.16%。水利建设如火如荼。中南部城乡饮水安全水源和连通工程有序推进，中庄水库坝体工程完成填筑总量的72.4%;龙潭水库建成，控制性工程大湾隧洞全线贯通;彭阳县安家川片区和西吉县葫芦河片区连通工程建成通水，22.4万群众提前喝上安全水。完成10处农村安全饮水、20座水库除险加固工程。治理水土流失面积327.3平方公里。原州区、彭阳县获全区农田水利建设二等奖。公路建设量大面广。自治区60%的交通项目在我市实施，东毛、固西高速和彭青、西毛一级公路加快建设;福银高速固原南出口、固胡路、国道309河川至固原等公路开工建设;南雁路下穿福银高速公路桥、东环路、北环路、国道309改线等道路建成通车，形成首条市区环城路;建成农村公路500公里。电力、通信等工程有重大进展、新的突破。生态环境更加美丽。全面实施主干道路大整治大绿化、月亮山水源涵养生态修复二期、移民迁出区生态修复、矿山环境治理和高标准基本农田建设等项目，完成营造林61.7万亩，森林面积达到339万亩，森林覆盖率达到21.4%。国家三北工程黄土高原综合治理林业示范建设现场会在彭阳县召开。环境污染综合治理效果明显，残膜回收率达到84.06%。绿色生态成为固原的靓丽名片。

　　——民生改善提标扩面。坚持民生优先、民生为本、惠民为要，全市民生支出占财政总支出80%以上，20件民生实事全面完成。2013年小康实现程度68.05%。扶贫开发稳中有进。实施精准扶贫，对全市624个贫困村36.14万贫困人口建档立卡。整合资金15.1亿元，实施5个整乡、125个重点贫困村整村推进工程，实现63个重点贫困村脱贫销号，减少贫困人口7万人。彭阳县获全区扶贫开发工作一等奖，原州区、泾源县获二等奖。闽宁协作进一步深化，在闽宁第十八次联席会议期间成功签订产业园区建设、特色农业种植、食品加工、文化旅游等合同项目11个，总投资60.5亿元。生态移民成效显著。全面完成“十二五”县内移民任务，累计搬迁1.8万户7.96万人;移民新村基础配套、产业培育、社会治理等同步推进。搬迁安置县外移民7457户3.25万人，累计2.44万户10.74万人，完成“十二五”计划的72.5%。隆德县获全区生态移民工作2013年一等奖、2014年二等奖。社会事业全面进步。办好人民满意教育，积极构建高效课堂，高考本科一次性上线率高出全区平均水平4.29个百分点，市民族职业技术学院顺利通过国家首批中职改革发展示范校建设验收;新建、扩建学校30所，改造332所学校运动场、食堂和供暖设施，新增基础教育学位6000个;全面落实教育惠民政策，建立从学前到高等教育全覆盖的资助体系。实施文化惠民工程，新建乡(镇)文化站、社区多功能运动场7个，创排《花儿醉六盘》等精品文艺节目15个，开展“首届全民健身月”等群众体育活动。深入推进医药卫生体制改革，全面推行“先住院、后付费”诊疗服务模式，新建、改扩建乡(镇)卫生院10所、社区卫生服务站10所。落实长效节育措施14618例，实施“少生快富”3184例，全区人口和计划生育工作现场观摩会在我市召开。率先在全区开展城乡居民大病医疗保险试点，受益面达到90%以上。新建公租房3458套，改造城市棚户区1.26万户、农村危房危窑3.5万户。城乡低保管理进一步规范，新建敬老院7所、农村幸福院120个、城镇养老服务站26个，泾源“老饭桌”模式在全区推广。实施法律援助民生工程1132件，受益群众1715人。救助各类残疾人4万人，实施康复服务4500人。发放创业小额担保贷款4.94亿元;创造就业岗位6610个，实现城镇新增就业7513人;培训各类农村实用人才2.9万人;农村劳动力转移就业30.7万人，实现工资收入41.7亿元。

　　一年来，突出抓了以下工作：

　　一是创新方法保增长。坚持稳中求进总基调，精准发力，综合施策。聚焦政策抓谋划。紧盯国家产业政策和自治区产业优化升级方向，高标准编制并经自治区批复了《全市产业结构调整和优化升级实施方案》;争取自治区召开第五次固原工作会，出台了《关于深入实施扶贫攻坚战略加快西海固地区经济社会发展的若干意见》，明确了8个方面25条扶持政策。明确责任抓落实。将全市主要经济指标、重点项目、重要工作、民生实事、深化改革重大任务量化分解，责任到人，实时监测调度，确保按时序节点和进度要求推进落实。现场协调抓进度。扎实开展“转作风、抓发展”活动，坚持一线作战，破解难题，促开工，抓质量。全面落实“一企一策”措施，驻企帮扶，跟踪服务，支持工业企业挖潜改造、提质增效。强化督查督办，实行明察暗访、定期通报，促进各项目标任务落实。

　　二是争取支持调结构。多措多方落实资金，优化投资结构和产业结构，三次产业结构调整为23.2：27.8：49。争取国家、自治区各类补助资金148.1亿元，增长15.7%。加强政银企对接，争取金融支持，年末金融机构存贷款余额分别达到313.14亿元、205.44亿元，贷存比达65.61%，提高5.79个百分点。市财政安排6000万元，扶持产业优化升级。成功举办了第六届中国(宁夏)园博会暨六盘山冷凉蔬菜节、宁蒙陕甘毗邻地区第五届经洽会和固原·福州经贸合作推介会等活动，实施招商引资项目176个，总投资495.78亿元，在工业项目引进上取得了突破。

　　三是依法治理创和谐。深入开展“六五”普法，推进宪法和法律“七进”活动。深化“平安固原”建设，严厉打击各类违法犯罪活动。完善排查化解社会矛盾和信访联动调解工作机制，积极调处各类矛盾纠纷，集中化解了一批信访突出案件。修订完善了各类应急预案，地质灾害预警及应急指挥系统建成使用。深入开展“企业安全生产主体责任年”活动，狠抓安全隐患排查整治工作，有效遏制重特大事故发生。实施食品药品安全示范创建工程，保障群众饮食用药安全。深入推进民族团结进步创建活动，实施“六个十”民族团结进步典型示范工程。全面开展宗教场所安全隐患排查整治工作，制定了宗教事务管理制度，规范宗教事务和活动，促进民族团结、宗教和顺。统计、人防、地震、气象、档案、地方志、老龄、妇女儿童、外事侨务等工作取得了新进步。

　　四是简政放权促改革。制定全面深化改革决定，明确“363”改革思路，40项改革任务全面完成。深化政府机构改革，加快政府职能转变。行政审批制度改革力度大、效果好，市级208项行政审批事项精简为57项，369项行政事业性收费项目精简为39项，减少收费1713万元。新注册企业1420家，增长82.05%。加快农村产权制度改革，试点林权抵押贷款，农村土地确权顺利推进。创新金融扶贫，设立1000万元担保基金，撬动信贷资金支持扶贫开发示范村建设，深受群众欢迎。制定廉租房、公租房并轨管理等制度，理顺市区共建共管等机制。改革投融资机制，引进社会资本参与城市基础设施、公益事业建设和经营。

　　五是转变作风树形象。深入学习贯彻习近平总书记系列重要讲话精神。扎实开展党的群众路线教育实践活动，推进各环节工作和各类专项整治行动，深化“三同六送六帮”活动，整改落实群众意见建议406条，整改“四风”突出问题49条。建成全市电子政务办公系统，政府会议减少23%，文件减少51%;“三公”经费下降12.5%;清理调整办公用房4.98万平方米。开展电视问政20期，开通政务微博，公开各类政务信息4364条，办理政民互动信件4527件。全面落实市委决定事项，办理区市人大代表议案、建议29件，政协委员提案163件，办复率100%。严格落实党风廉政建设责任制，加大对财政预算执行、扶贫救助资金、重大项目和财政专项资金审计监督，核减政府投资项目不合理资金1.04亿元;强化行政监察，严肃查办违纪违法案件，给予党纪政纪处分123人。认真落实党政主要领导不直接分管五项工作规定，制定落实公共资源交易管理办法、财政资金管理办法、政务公开制度、行政效能问责办法等制度，树立为民务实清廉良好形象。

　　各位代表，过去的一年，市政府坚持解放思想、改革创新，坚持依靠群众、群策群力，坚持改进作风、务实苦干，各项事业取得喜人成绩。这得益于自治区党委、政府和市委的正确领导和充分信任;得益于市人大常委会及代表、市政协及委员的有效监督和大力支持;得益于全市广大干部群众的积极参与和奋力拼搏。在此，我代表市人民政府，向各位代表和委员，向全市各条战线上的劳动者、各民主党派、工商联、人民团体、社会各界和中央、自治区驻固单位、人民解放军、武警官兵及公安干警，向所有关心支持固原建设和发展的朋友们，表示崇高敬意和衷心感谢!

　　各位代表，总结一年的工作，问题也需面对。一是产业支撑能力弱。农业品牌效应不明显，市场竞争力不强;工业骨干企业少，吸纳就业能力弱;第三产业内部结构不合理、增长慢，拉动需求不足，影响了地区生产总值未完成年增长12%的目标。二是重大项目推进慢。项目前期工作不充分，特别是中铝固原煤电铝一体化等3个重点项目未能开工;基础设施类投资比重高，产业类投资比重低，持续高速增长的后劲不足。三是扶贫开发压力大。县外移民受迁入地经济下滑等因素影响进展缓慢;部分贫困乡村道路不畅、饮水不净、住房不安等问题还比较突出;教育、卫生、社保等公共服务与群众期望还有较大差距。四是社会治理难点多。因利益诉求过高非访调处工作难度大，安全隐患整治任务重，治安防控体系盲点多，农村环境治理和城市社区物业管理等问题较为突出。五是作风转变任务重。有的部门和干部行政效能不高、工作作风不实，个别干部贪污、挪用扶贫资金、涉农资金等违纪违法行为时有发生。对上述问题，政府将以更大的决心、更硬的措施、更实的作风认真加以解决，履行好人民政府为人民的神圣职责，决不辜负全市人民的期望和重托!

　　2015年主要工作

　　2015年是全面完成“十二五”规划的收官之年，做好今年的工作尤为重要。我们要主动适应经济发展新常态，抢抓国家建设“一带一路”、支持民族地区发展的重大战略机遇，认真落实自治区加快西海固地区经济社会发展的政策，迎难而上，激情创业，务实苦干，推动发展。

　　今年政府工作的总体要求是：深入贯彻中央和自治区各项决策部署，全面落实市委三届六次全委会确定的目标任务和要求，坚持稳中求进总基调，以提高经济发展质量和效益为中心，以产业优化升级为支撑，以持续改善民生为根本，以重大项目建设为抓手，以深化改革为动力，以建设法治政府为保障，坚持推进扶贫攻坚和生态移民、工业强市、生态农业、文化旅游、新型城镇化、民生改善和社会治理战略不动摇。

　　主要目标是：地区生产总值增长10%左右，地方公共财政预算收入增长10%，地方公共财政预算支出增长10%，全社会固定资产投资增长20%左右，社会消费品零售总额增长12%左右，城镇居民人均可支配收入增长10%，农村居民人均可支配收入增长11%;城镇登记失业率控制在4%以内，居民消费价格指数涨幅控制在3%以内，人口自然增长率控制在10‰以内，完成自治区下达的节能减排任务，城市空气质量优良天数稳定在92%以上。

　　实现上述目标，重点抓好以下工作：

　　一、创新扶持机制，推动产业升级再上新水平

　　落实产业优化升级方案，统筹安排资金1亿元，助推优势特色产业提质增效，增强综合经济实力。

　　培育升级优势工业。统筹安排4000万元，扶持能源、环保新材料、农产品加工、先进制造业健康发展。完善盐化工示范区、轻工产业园和县(区)产业园区基础配套，提升园区承载能力，新引进企业20家。创新与发达地区、市县共建产业园区的新机制。争取开工建设中铝固原煤电铝一体化、欣正天然气液化、海尔电子厨具等项目;加快推进金昱元盐化工产业链、王洼煤矿千万吨级矿区、申科电力设备生产、福宁广业马铃薯淀粉深加工、西吉五瓣兰胡麻油生产、彭阳轩峰20万吨饲料加工等项目;力促家道全谷物食品加工、国电固原风场300兆瓦工程、天润风电二期、西吉佳立马铃薯淀粉厂迁建、隆德新坐标服装加工等项目建成投产;开展六盘山热电厂二期扩建前期工作。认真落实加快发展非公有制经济各项政策措施，鼓励技术创新和管理创新，支持中小微企业技术改造，加快“两化”融合，推进传统加工业向中高端迈进。年内新增规上企业7家。工业增加值增长20%。

　　壮大升级特色农业。统筹安排3000万元，发展壮大马铃薯、肉牛养殖、冷凉蔬菜、中药材、苗木产业。依托国家农业科技园区，建成一批现代农业生产示范基地，加快推广应用新品种、新技术、新装备。抢抓实施马铃薯主粮化战略机遇，巩固扩大种薯三级繁育体系，原原种繁育能力达到1.6亿粒，种薯繁育面积稳定在28万亩以上，提高马铃薯单产和品质。扶持基础母牛、养殖示范乡村、专业大户和优质饲草料基地建设，与澳大利亚洛克汉普顿(ART)基因公司合作建设牛胚胎移植中心，发展万头肉牛养殖示范乡30个、千头肉牛养殖示范村166个，千头肉牛养殖场10个，新增肉牛5万头，饲养量达到105万头;争取中国肉牛产业发展大会在固原召开。创建种植业示范园区50个，冷凉蔬菜种植面积稳定在58万亩以上;中药材达到15万亩;苗木稳定在40万亩以上。积极与金融机构协作，推行蔬菜、肉牛价格政策性保险。培育大型农产品流通企业3家、农民专业合作社100家、家庭农场20家，组建六盘山生态农产品联盟，打好绿色牌，提升农产品核心竞争力。农产品加工转化率达到48%。农业增加值增长5%以上。

　　拓展升级文化旅游业。统筹安排3000万元，支持文化与旅游深度融合发展。启动建设市域内旅游环线，开工建设六盘山国际旅游休闲度假区和六盘山红军长征景区创建5A级提升改造项目，加快黄铎堡、火石寨等旅游特色镇建设，完成泾河源旅游小镇二期工程，开发乡村旅游休闲产品;整合旅游资源，积极推进六盘山森林公园与老龙潭联合运营、须弥山与火石寨一体经营，与周边市合作，打造六盘山大旅游圈;进一步加大宣传，规范管理，提升服务，力争全年接待游客275万人次，实现旅游社会收入12.1亿元。抓紧推进“丝绸之路”固原段申报世界文化遗产工作，实施北朝——隋唐墓地、须弥山石窟、开城遗址文物保护工程，开工建设隋唐历史文化园。加快市文化馆、图书馆、艺术剧院建设，积极推进中华民俗文化园项目早日开工。出台促进服务业发展意见，加快建设西兰银综合物流园、六盘山(国际)农产品物流中心和各县(区)专业批发市场，积极发展金融保险、电子商务、物业管理、快递服务、信息咨询等新型服务业态。服务业增加值增长8%。

　　二、创新投入机制，推动民生改善取得新成效

　　聚焦扶贫攻坚主战场，全面落实各项惠民政策，坚持民生投入只增不减，切实办好15件民生实事，让群众真正享受到改革发展的成果。

　　创造更多的致富机会。继续加大精准扶贫力度。坚持整体推进与精准到户相结合，危房危窑改造与美丽乡村建设相结合，实施扶贫整乡推进乡(镇)11个，创建扶贫整村推进示范村30个;完善专项扶贫、行业扶贫、社会扶贫“三位一体”大扶贫格局;深化闽宁对口扶贫协作，力促闽宁第十八次联席会议签订的项目落地实施;扩大金融支持扶贫示范村建设规模，发挥好担保基金的撬动作用;完成126个重点贫困村销号，减少贫困人口7万人以上。全面完成生态移民任务。切实加强移民新村管理，推进移民新村后续产业培育发展，确保移民近期生活有保障、长远致富有门路;加强县外移民对接沟通，完成剩余9792户40841人的搬迁工作，全面完成“十二五”移民任务。多渠道开发就业岗位。大力实施“就业创业五项计划”，以创业促就业，以就业促增收，发放创业小额担保贷款3.7亿元以上，发展小企业400个，培养小老板900人，创造就业岗位4000个，新增城镇就业7000人;加快劳务产业转型升级，落实农民工职业技能提升计划，维护农民工权益，转移农村劳动力就业30万人以上，实现工资总收入45亿元以上。

　　提供更好的公共服务。优先发展教育事业，启动实施第二期学前教育三年行动计划，新建幼儿园19所;加快义务教育学校标准化建设，完成义务教育薄弱校校舍改造10万平方米，原州区、彭阳县年内通过自治区义务教育均衡发展评估验收;大力发展普通高中教育，合力推进职业教育;在西南新区新建1所小学、1所幼儿园，启动建设市第八中学，完成固原二中、回中、一小、实验小学扩建及市幼儿园迁建等工程，扩大优质教育资源，缓解城区中小学“大班额”和幼儿入园难问题;加强教师队伍建设，提高教育教学质量;落实各项教育惠民政策，惠及21.3万名学生。支持宁夏师范学院提升办学质量和水平，为固原培养更多高层次实用人才。加快乡(镇)、村、社区文化场所建设，完善公共文化服务体系;深入实施全民健身工程。积极开展爱国卫生运动;推动医改向纵深发展，完善“先住院、后付费”诊疗服务模式，加强村医队伍建设，落实基本公共卫生服务和健康促进计划;建立全市120急救指挥平台;加强重大疾病防控工作。完善计生优质服务体系和利益导向机制，实施少生快富工程3000例以上。新建公租房1632套，改造城市棚户区7967户、农村危房危窑3万户。加强社区建设和管理，新建城乡社区服务站31个，提升社区服务水平。加快城市养老城建设，新建农村幸福院117个、农村敬老院10个;建成回汉公墓。认真落实老干部政策待遇，加强老干部活动场所建设。加强残疾人基本民生保障，残疾人救助和康复服务覆盖面达到80%以上。完善社保经办管理体制，推进全民参保登记和扩面征缴，落实失地农民养老保险政策，提升社会保障能力;加强城乡低保规范化管理和社会救助，构筑困难群众基本生活保障线。狠抓“米袋子”“菜篮子”工程，出台扶持配套政策，完善市场体系建设;严格市场监管，严厉打击哄抬物价等违法行为，确保物价稳定。

　　打造更优的社会环境。深化“平安固原”创建工作，落实和发挥基层组织在社会管理方面的关键作用，不断拓宽公众参与社会治理的渠道，深化矛盾纠纷排查调处工作，妥善化解信访积案。加强精神文明和民风建设，使社会主义核心价值观成为广大群众的价值取向和日常行为。依法打击各类违法犯罪活动，加快立体化社会治安防控体系建设，实施网格化管理、社会化服务，不断提高人民群众的安全感和满意度。健全网络舆论引导及违法犯罪预防机制，净化网络空间。强化政府应急保障能力建设，重视预警监测和风险评估，有效应对自然灾害、事故灾难等突发事件。落实企业主体安全生产责任，有效防范和遏制重特大安全事故。强化食品药品监管，确保群众饮食用药安全。深入开展民族团结进步创建活动，使“两个共同”“三个离不开”“四个认同”思想在各族群众心中深深扎根。依法加强宗教事务和宗教活动管理，严厉打击非法宗教渗透活动;抓好宗教人士和教职人员培训教育，充分发挥宗教界人士在化解矛盾纠纷中的积极作用，促进宗教领域和谐稳定。加强档案、统计、地震、人防、气象、地方志、外事侨务等工作。支持工会、共青团、妇联等人民团体开展工作。

　　三、创新保障机制，推动项目建设实现新突破

　　规划一批重大项目引领科学发展，落实工作责任，实施好60个重点项目。

　　抓好规划编制。抓住新机遇、发挥新优势、用好新政策、借助新力量，编好“十三五”规划纲要和专项规划。围绕产业升级、基础设施、扶贫攻坚、民生改善、公共服务、生态治理等重点领域，主动谋划、研究论证一批事关全局和长远发展的大项目、好项目，争取更多的项目纳入国家、自治区“十三五”规划。认真落实《宁夏空间发展战略规划》，高质量完成产业发展、城乡建设、国土利用等 “三规合一”编制工作，指导经济社会全面协调可持续发展。

　　建好重点项目。继续实施好东毛、固西高速和国道344线青石嘴——泾源、省道203线王洼经彭阳——高寨塬、福银高速固原南出口等公路建设，建成原州区——王洼铁路、彭青、西毛一级公路和国道309河川——固原段，开工建设泾源——隆德山河、隆德——庄浪、西吉——三合、固将公路张易——将台段等干线公路，新建乡村公路500公里，形成全市1小时交通圈;做好固原——石嘴山城际铁路、固原火车站改扩建、白银——隆德——平凉铁路、彭阳——庆阳高速、隆德——张易公路等项目前期工作;争取开通固原——北京、固原——东南沿海等地区的旅游航线。加快推进中南部城乡饮水安全水源及连通配套工程建设，力争主体工程全面完工;完成5处农村安全饮水工程;积极实施抗旱应急工程;完成盐化工示范区供水和贺家湾、沈家河等34座中小型水库提升改造、除险加固工程;争取开工建设彭阳李渠等2座水库;治理水土流失面积300平方公里。开工建设太阳山——六盘山——平凉750千伏输变电工程，建设彭阳白阳110千伏输变电工程和清水河330千伏变电站扩建工程，提升供电网络保障能力。

　　做好服务保障。组织开展“准备之冬、开工之春”活动，提前做好项目土地报批、征地拆迁、规划设计和招投标等前期工作，实行项目包抓负责、协调联动机制，争取新建的33个重点项目上半年全部开工建设。争取国家、自治区各类补助资金145亿元，融资贷款27.5亿元，其中国家开发银行宁夏分行棚户区改造中长期贷款20亿元，争取发行债券7.5亿元。争取国有商业银行新设分支机构，石嘴山银行固原分行开业，组建村镇银行1—2家。创新金融政策，力争金融贷存比达到70%以上。支持鼓励社会资本参与城乡建设。加大招商引资工作力度，健全招商引资项目落实机制，加强协调服务，力争实际到位资金150亿元以上。

　　四、创新建管机制，推动城乡建设展现新面貌

　　牢固树立“绿水青山就是金山银山”的理念，坚持硬件建设和软件管理两手抓，保持天蓝、地绿、水清、城净的优美环境。

　　高品质建设城市。开展国家新型城镇化综合试点，用好支持政策，引导农民变市民，加快新型城镇化进程，城镇化率达到43%。按照《宁夏空间发展战略规划》，结合清水河城镇产业带布局，加快宁南区域中心城市建设，西南新区市医院、九龙佳苑、安置房等工程竣工投入使用;建成博物馆应急避难广场、儿童公园等项目，加快清水河国家湿地公园、北海湿地公园、饮马河城市休闲森林公园和东岳山生态文化园建设;新建、改造城市道路(街巷)28.8公里;完成城市生活垃圾填埋场二期和南塬水厂一期工程;配套给排水、供热管网94公里，新建标准化菜市场4个、公厕10座、城市集中停车场3个，增强中心城市集聚承载力和辐射带动力。推进大县城建设，实施西吉县体育馆、隆德县博物馆、泾源县城市防洪贯通、彭阳县城东环路等工程，完善综合服务功能。加大房地产和建筑市场监管力度，进一步规范建筑市场。

　　精细化管理城市。持续开展城乡环境综合整治，深入推进“五城联创”，对照创建标准，定任务、定责任、定时限，推进硬化、净化、亮化、绿化、美化 “五化”工程;积极开展群众性的主题创建活动，引导广大市民爱我城市，争做文明市民;下决心解决市区文化街交通拥堵、秩序混乱和部分道路路口交通安全隐患问题，使市容更整洁、景观更宜人、出行更安全、城市更宜居。争取将我市列入国家“智慧城市”建设试点城市，引入智慧城市管理架构，建成数字化城市管理平台;加强城市管理综合执法机构建设，实施精细化、网格化管理，提高城市管理水平。年内创建成自治区卫生城市和双拥模范城市。

　　全方位打造美丽乡村。实施黄铎堡、火石寨、杨河、六盘山、古城等9个中心镇和25个美丽村庄建设，完善农村水、电、路、网和垃圾处理等公共基础设施，打造特色明显、宜业宜居的美丽乡村。加强耕地保护，坚决制止违法乱建行为。大力推进大六盘生态长廊保护工程，实施新一轮退耕还林和水源涵养林建设等重点项目，完成营造林30万亩，其中人工造林20万亩、封山育林10万亩。完成移民迁出区生态修复20万亩。把植树造林作为固原生态建设的“一号任务”坚持不懈抓下去，加大退耕还林抚育管护和禁牧封育工作力度，抓好六盘山国家级自然保护区和各县(区)国有林场的经营管护，巩固生态建设成果。森林覆盖率提高0.8个百分点。继续开展主干道路大整治大绿化工程，完成彭青、G309、S202和S203等道路绿化带建设。持续推进农村环境集中连片整治示范项目，加大重点污染源监测治理力度，切实解决清水河、葫芦河、渝河等流域水污染问题;抓好节能减排、关停落后产能、残膜回收等工作，净化美化家园。

　　五、创新服务机制，推动依法行政迈出新步伐

　　认真贯彻落实市委全面推进依法治市的实施意见，加快服务型法治政府建设。

　　厘清责任推进依法行政。依法全面履行政府职能，推进机构、职能、权限、程序、责任法定化。建立行政权力审批清单、权力运行责任清单和市场准入负面清单“三个清单”制度，做到法定职责必须为，法无授权不可为。健全依法决策机制，把公众参与、专家论证、风险评估、合法性审查、集体讨论决定确定为重大行政决策法定程序，落实政府法律顾问制度，确保决策制度科学、程序正当、过程公开、责任明确。深化行政执法体制改革，完善市、县(区)两级政府行政执法管理，健全“两法衔接”工作机制，推进综合执法和跨领域执法。完成“六五”普法，启动“七五”普法。积极推进覆盖城乡居民的公共法律服务体系建设，加强公务人员法律培训，提高法治思维和依法行政能力。健全依法行政监督体系，主动接受人大法律监督，认真执行人大及其常委会的决议，定期报告工作，配合做好工作视察;自觉接受政协民主监督，认真听取各民主党派、工商联、无党派人士和人民团体的意见，及时通报情况，支持政协落实协商工作计划;坚持与人大代表、政协委员联系协商制度，办理好人大代表议案建议和政协委员提案;广泛接受社会监督、舆论监督，重大决策事项邀请市民代表参与，并及时向社会公布，充分发挥媒体在推进工作中的监督作用。建立健全以网络、短信为主的政务公开网络平台，推进决策、执行、管理、服务和结果“五公开”，让权力在阳光下运行。

　　落实制度规范政府行为。巩固提升教育实践活动成果，建立制度化、规范化长效机制。深化制度创新和管理，落实公共资源交易、财政和扶贫资金、政府投资项目、政府性资金审计监督、城市规划建设管理、政务公开和行政效能问责等16项制度，用制度管事管权管人，特别要加强扶贫资金、涉农资金、民生资金和重点项目的监督管理。教育引导行政人员树立法治意识、规矩意识和纪律意识，提高制度执行力，对违反制度规定的行为实行零容忍，维护制度法纪的严肃性。

　　解放思想深化改革开放。继续深化“思想大解放、树立新形象”活动，破除思想观念束缚，推进体制机制创新，向改革开放要动力、要活力。按照“放权、减负、创新”的要求和我市深化改革30条，全面推进46项改革任务，着力打造“两优”环境。加快政府职能转变，进一步简政放权，再取消和下放一批行政审批事项，全面清理非行政许可事项;推进政务服务标准化建设，优化审批办事流程，探索建立提前评估、预先许可的预审批制度，加快推进部门行政审批事项集中审批或授权进驻大厅审批。坚持放管结合，完善市区共建共管机制。深化经济体制改革，完成农村土地、房屋确权颁证，开展农村集体资产股份制、农村产权流转进入公共资源交易中心试点工作;推动中小企业私募债发行和林权、养殖产权抵押贷款试点工作;积极推进水资源管理体制及水价改革，提高水资源利用率;深化投融资改革，完善贷款担保融资机制，建立信用监测、评价和惩戒体系，完善社会征信系统。启动机关事业单位养老保险并轨改革和公车改革。推进户籍制度改革。创造有利于对外开放的良好环境，主动融入“两区”建设和丝绸之路经济带战略支点建设，围绕中阿博览会，探索建立交流合作机制，推动商贸、文化、旅游等领域务实合作，提升对外开放水平。

　　勤政廉洁促进作风建设。认真落实“八项规定”，深入践行“三严三实”要求，严格落实党风廉政“一岗双责”，全面推进惩治和预防腐败体系建设。严守党的政治纪律和政治规矩。进一步改进文风会风，政府会议、文件再分别精简15%以上。进一步改进办公方式，全面推行电子政务办公系统，降低行政成本，实现市级“三公”经费零增长。严格预算刚性约束，加大政府债务偿还和化解力度，有效防控债务风险。统筹抓好政府系统党的建设、干部教育和人才队伍建设等工作。深化“三同六送六帮”活动，帮助群众解决实际困难和问题。深入推进政府系统效能建设和群众评议机关干部作风活动，深化政风行风热线、电视问政和微博、短信问政、问需、问计等活动，提高行政效率。加强审计监督和行政监察，加大行政效能问责力度，严肃查处损害群众利益的人和事，以反腐倡廉的实际成效聚民心、转作风、促发展。

　　各位代表，新常态要有新担当，新任务要有新作为。让我们紧密团结在以习近平同志为核心的党中央周围，在自治区党委、政府和市委的坚强领导下，解放思想，坚定信心，创新实干，全面完成“十二五”规划目标任务，为建设固原和小康社会而努力奋斗!
